# Directed aging, memory, and nature’s greed

**DOI:** 10.1126/sciadv.aax4215

**Published:** 2019-12-20

**Authors:** Nidhi Pashine, Daniel Hexner, Andrea J. Liu, Sidney R. Nagel

**Affiliations:** 1 Department of Physics and The James Franck and Enrico Fermi Institutes, University of Chicago, Chicago, IL 60637, USA.; 2 Department of Physics and Astronomy, University of Pennsylvania, Philadelphia, PA 19104, USA.

## Abstract

Disordered materials are often out of equilibrium and evolve very slowly in a rugged and tortuous energy landscape. This slow evolution, referred to as aging, is deemed undesirable as it often leads to material degradation. However, we show that aging also encodes a memory of the stresses imposed during preparation. Because of inhomogeneous local stresses, the material itself decides how to evolve by modifying stressed regions differently from those under less stress. Because material evolution occurs in response to stresses, aging can be “directed” to produce sought-after responses and unusual functionalities that do not inherently exist. Aging obeys a natural “greedy algorithm” as, at each instant, the material simply follows the path of most rapid and accessible relaxation. Our experiments and simulations illustrate directed aging in examples in which the material’s elasticity transforms as desired because of an imposed deformation.

## Teaser

Plastic deformation produced by external strains directs the aging of disordered materials to create unusual elastic response.

## INTRODUCTION

The incremental process of aging affects materials over extended periods of time (*1*–*4*); for example, a plastic may become more brittle or a glass may slowly shrink in volume. These changes occur without the apparent influence of an external force directing the evolution. They are simply part of the inexorable aging process, which often leads merely to a degradation of physical properties. However, it comes as no surprise that in other cases, such as a beam sagging under the influence of gravity, an externally imposed stress forces the material to deform over time in an obvious and well-defined manner. Here, while the geometry of the beam has perceptibly changed so that it may no longer do the job for which it was originally intended, changes in the material’s elastic properties are less apparent.

Our purpose here is to highlight how the mechanics of materials can evolve in unexpected ways due to a process of directed aging. Materials that have not reached equilibrium often retain a memory of how they were processed, trained, stored, or manipulated (*5*). During aging, they evolve in a direction dictated by those conditions. The resulting properties contain a memory of the applied load. We unite these concepts of directed aging and memory with the idea that nature often follows a greedy algorithm in its evolution. We first recall how materials can be manipulated on the computer and then explore how natural evolution can be harnessed to produce a similar outcome.

Materials can often be considered on an atomic scale as a network of bonds between nodes (*6*, *7*). Previous work has shown that networks can easily be tuned to have specific properties by removing a small subset of computationally determined links between nodes. For example, a spring network can be transformed from being nearly incompressible with a positive Poisson’s ratio, ν ≈ 0.5, to being almost completely auxetic with ν ≈ −1 (i.e., so that compression along one axis causes the transverse directions to become equally compressed) (*8*–*11*); the network can be tuned over the entire spectrum of elastic behavior into a regime where few materials exist. Likewise, networks can be pruned for allosteric behavior so that a local applied strain induces a large displacement at a distant point (*12*–*14*).

It takes unexpectedly few alterations in the original network to create either of these functionalities. A takeaway message is that structures generated from packings with rugged energy landscapes are often extremely malleable—with seemingly modest modifications, they can be easily manipulated to have unusual, esoteric, and finely tuned properties.

In these examples, a “greedy” computer algorithm was used at each stage to find the alteration (i.e., pruning of a bond) that brings the network closest to the desired final state. The algorithm does not require—nor necessarily benefit from—a more sophisticated evolutionary process that samples many alternative paths to reach an optimal outcome.

Here, we explore the extent to which a material in the process of aging can be considered as following nature’s—as distinct from a computer’s—greediness to achieve unconventional properties. Can a material, without the intervention of a computer, transform by retaining a distinct memory of the forces it has encountered in its lifetime?

### Gedanken experiment

We start with a highly idealized gedanken experiment on a large heap of sand. Grains in this heap are under pressure from the material above it. Deep in the pile, the pressure is enormous and some of the contacts between grains experience immense forces. As they age, it is reasonable to expect that the contacts deform plastically, with those experiencing the largest forces deforming most rapidly. Over long times, these incremental deformations could become substantial and change the contacts between grains considerably. While this seems straightforward, this system is interesting because there is preferential alteration of the bond characteristics depending on the magnitude of stress that each individual contact feels under the applied stress.

To gain insight into the effect of such preferential alteration, we recall the evolution under selective bond pruning of a spring network under compression. An idealized example of frictionless spherical grains jammed by compression (*15*) can be converted into a network by replacing spheres with nodes and replacing contacts between spheres with unstretched springs connecting the nodes (*16*, *17*). In such disordered networks, the contribution of any specific bond to the bulk modulus, *B*, is, to a large extent, independent of its contribution to the shear modulus, *G* (*8*). If bonds are pruned according to how much stress they feel because of an externally applied stress, the system’s bulk and shear moduli change in different ways. In particular, when the system is placed under isotropic compression, pruning of bonds under the largest stress causes *B* to decrease more rapidly than *G* so that the ratio *G*/*B* increases (*8*). Note that we prune a bond according to the stress it is under and not according to how much it would change a modulus if it were removed. While the latter is a more effective way to prune a network (*9*, *10*), it is not essential.

For an isotropic material, the Poisson’s ratio, ν, is a monotonic function of (*G*/*B*): $\nu =\frac{d-2(G/B)}{d(d-1)+2(G/B)}$, where *d* is the spatial dimension. Therefore, as (*G*/*B*) increases, the material is driven to have a negative Poisson’s ratio. This result for pruned networks suggests that a sandpile under pressure could evolve toward auxetic behavior as well—an unexpected outcome.

Note that our gedanken experiment neglects any particle rearrangements that could occur during aging (*4*, *18*, *19*) and is therefore valid only at the early stages before particle rearrangements occur. The experiments and simulations we describe below also do not allow particle rearrangements.

## RESULTS

### Results from experiment

**Fig. 1:**
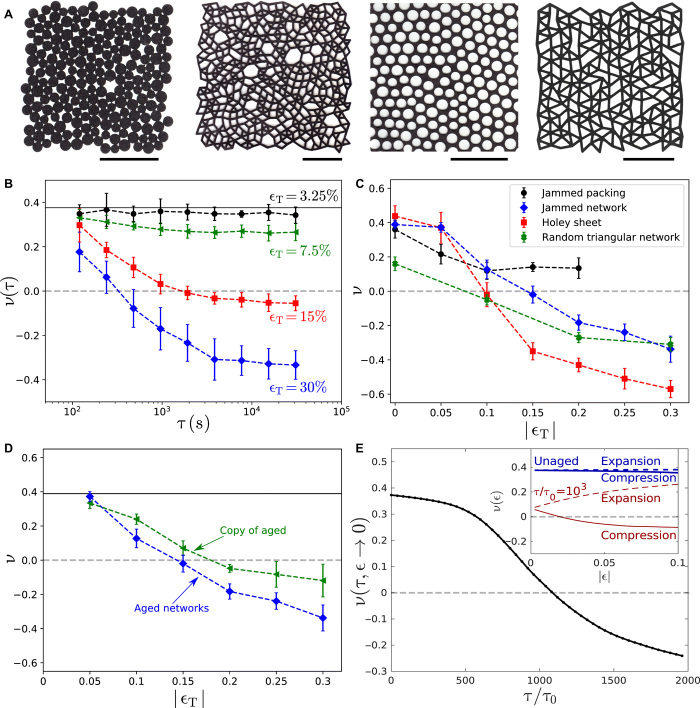
Change in Poisson’s ratio, ν, by aging. (**A**) Sample systems of each kind that were trained. Left to right: a jammed packing of discs, a network based on jamming, a disordered holey sheet, and a random network based on triangular lattice. Scale bars, 50 mm. (**B**) Effect of aging as a function of time (seconds). When foam networks are aged under constant strain, the Poisson’s ratio drops until it saturates to a final value. Different curves represent different aging strains. The horizontal line at 0.38 shows the initial ν of the networks. (**C**) Four different systems: jammed packings (black circles), jammed networks (blue diamonds), holey sheets (red squares), and random triangular networks (green crosses) show a drop in ν when aged under uniform compression. (**D**) Role of geometry: Networks prepared by aging under different strains (blue diamonds) compared to unaged networks cut out to have the same geometry (green squares). The deviation suggests that both geometry and the material properties change during aging. (**E**) Results from numerical simulation of a system aged at 2.5% compressive strain. The Poisson’s ratio within linear response decreases as a function of time. Inset: The Poisson’s ratio in the nonlinear regime as a function of the measuring strain for unaged networks and networks aged at τ/τ_0_ = 10^3^. Note that the system is not auxetic within linear response but auxetic when compressed to larger strains.

The gedanken experiment inspires the following experiments on aging under compression. From a sheet of EVA (ethylene vinyl acetate) foam, we laser-cut two-dimensional (2D) systems as shown in Fig. 1A. We make four different kinds of systems as described in Materials and Methods: jammed packings of discs, networks derived from jammed packings, random holey sheets, also derived from jammed packings, and random networks based on a triangular lattice.

All of these systems are then aged by confining them for a time τ in a square rigid box that has a smaller edge length, *L*_box_, than the original length of the system, *L*_initial_. We define the training strain as ϵ_T_ ≡ (*L*_box_ − *L*_initial_)/*L*_initial_. Since we train our samples under compression, our values for ϵ_T_ are always negative. We measure the Poisson’s ratio, ν(τ, ϵ_T_), by removing our sample from the confining box and immediately compressing it along one axis while measuring the deformation in the perpendicular direction (see Materials and Methods). Figure 1B shows data for the Poisson’s ratio of networks as a function of training time, τ. For small training strains, we do not see any substantial change in ν, but for ∣ϵ_T_∣ ≥ 0.15, ν eventually becomes negative. This suggests that aging here is a nonlinear effect that requires large strains. Figure 1C shows how ν changes on aging for different values of imposed strain in the long-time limit. For all four systems, ν decreases in accord with the reasoning behind the gedanken experiment. For the jammed networks, random triangular networks, and holey sheets, ν becomes negative at larger values of ∣ϵ_T_∣.

We note that the decrease in ν is different in each of the four systems. Our data suggest that systems with a larger void fraction (fraction of material that has been cut out) reach a smaller Poisson’s ratio. A correlation between density and the Poisson’s ratio, ν, has been reported in (*20*). We note, however, that previous work on networks showed that the Poisson’s ratio could be varied between its two extreme limits while keeping the number of network contacts (and therefore the network density) fixed (*8*). In keeping with this result, the Poisson’s ratio of our systems before aging does not depend strongly on the void fraction. Only after aging under compression do we find a correlation. This suggests that the low-density networks have a higher capacity to be trained. Perhaps large voids allow larger nonaffine strains in the network, which, in turn, produce larger changes in the network structure and stiffness.

In this example of directed aging, the material naturally acquires an auxetic response. The fact that all systems show similar behavior suggests that directed aging of isotropic disordered systems under compression may lead more generally to reduced values of the Poisson’s ratio.

There are two ways in which aging may have affected the network: (i) bond strengths change due to the stresses to which they were exposed, and (ii) the geometry of the network changes due to internal rotation, deformation, and/or buckling of bonds. To assess the relative contributions of these two effects, we image an aged network. We use that image to pattern another network out of unaged material that is as geometrically identical as possible to the aged one. The difference between the Poisson’s ratio of these two (nearly) identical networks is presumably due solely to the aging of the stiffness of the contacts.

The Poisson’s ratio of these geometrically identical but unaged networks is shown in green triangles in Fig. 1D. We consistently find that the unaged, copied networks have a lower Poisson’s ratio than the original networks but not as low as that of the aged ones. This implies that some of the contribution to the aging process derives from a change in the network geometry and some is due to a change in the material stiffness.

Our experimental protocol is similar to earlier work by Lakes (*21*, *22*), in which foam was made auxetic by heating it while under compression. In that work, the geometry of the structure was observed after the foam was returned to ambient temperature. The evolution of auxetic behavior was attributed to the creation of concave polygons, which are known to decrease the Poisson’s ratio of a material (*11*, *22*, *23*). In contrast, on aging a bulk piece of our EVA foam under compression, the Poisson’s ratio does not decrease but rather remains constant or increases slightly. We find that aging decreases the Poisson’s ratio only when voids or patterns are cut out, suggesting that the scale at which the material changes is not at the microscopic scale but rather at the much larger scale of the network bonds. This suggests that our particular choice of foams is not essential and that other materials that undergo plastic deformation would yield similar behavior. We find a similar decrease in the Poisson’s ratio upon aging networks made of 3D printed polyurethane. Our results of Fig. 1D also show that geometrical changes are not the only contribution to the decreased value of ν; changes in bond stiffness also participate in creating auxetic behavior.

### Results from simulations

To further explore how changes of bond stiffnesses under stress can affect the Poisson’s ratio, we conduct simulations of a simple model of aging spring networks. We start by considering a network in which each spring, *i*, has spring constant *k_i_* and an unstretched length $l_{i}^{0}$. When compressed, the spring has length *l_i_*. Because of disorder, each spring will generally be compressed by a different amount. The energy of the resulting network is the sum of the energies of all the springs

$$E=\frac{1}{2}\sum_{i}k_{i}{\left(l_{i}-l_{i}^{0}\right)}^{2}$$ (1)

For simplicity, we have omitted the energy due to bending angles around a node (*11*).

Now, consider two limiting cases. The first corresponds to the case in which aging is due completely to the evolution of the bond strengths *k_i_* under the imposed stresses. The second corresponds to aging arising completely from the evolution of the equilibrium distances between nodes, $\ell_{i}^{0}$. This is reminiscent of the geometrical mechanism proposed for foams by Lakes (*21*).

Here, we focus on the first case, deferring the second to a future publication. We evolve *k_i_* so that it decreases in time according to the elastic energy stored in that bond

$$\frac{{\mathrm{dk}}_{i}}{d\tau }=-\frac{1}{\tau_{0}{\bar{l_{i}^{0}}}^{2}}k_{i}{\left(l_{i}-l_{i}^{0}\right)}^{2}$$ (2)

The proportionality constant, τ_0_, and the average equilibrium length of the bonds, $\bar{l_{i}^{0}}$, are material dependent and set the units of time and length. Bonds under greater stress evolve more rapidly. At late times, *k_i_* → 0; we therefore consider Eq. 2 as an approximation valid only at early times. We evolve the system at a prescribed training strain, ϵ_T_, and then measure the elastic response with respect to the zero-strain state, which remains the global energy minimum. We assume that *k_i_* does not evolve during the measurement process itself.

Figure 1E shows the evolution of the Poisson’s ratio ν calculated at ∣ϵ_T_∣ → 0, when the system is aged under compression. Evidently, ν decreases in time, consistent with the experimental data in Fig. 1C and eventually becomes negative. The aging evolves in a directed manner—under compression, the bulk modulus *B* decreases with respect to the shear modulus, *G*, leading to an auxetic material.

This model also predicts interesting nonlinear behavior. The nonlinear Poisson’s ratio is defined by ν(ϵ) = −ϵ_r_/ϵ, where ϵ is the imposed strain along one axis and ϵ_r_ is the resulting transverse strain after minimizing the energy with respect to the locations of all the nodes and the box shape. The inset of Fig. 1E shows ν(ϵ) for the unaged network and for a network that has been aged until τ/τ_0_ = 10^3^. The original, unaged network depends only weakly on the measuring strain ϵ, even up to 10% strain. For aged networks, however, the nonlinear Poisson’s ratio, ν(ϵ), depends on ϵ, the strain at which it is measured, as shown in Fig. 1E. The material can become auxetic even when it is not auxetic in linear response. In other words, compressing the system along one axis leads to transverse expansion at small strains and to transverse compression under larger strains. This nonlinear behavior is difficult to achieve by design but, here, occurs naturally.

Both the experiments and simulations demonstrate that aging under compression is directed toward a nontrivial elastic state with a lower Poisson’s ratio. The elastic properties start to change as soon as aging commences under the applied stress and evolve to be substantially different from those of the freshly prepared material.

### Directed aging under shear in laboratory experiments

Different aging protocols can evolve toward different limits. Here, we evolve a system under shear stress instead of compression. We return to the experimental networks described in Results from experiments, aging our networks under pure shear by compressing them in one direction and stretching them in the perpendicular direction. We measure the Poisson’s ratio in two perpendicular directions: (i) compressing the network first along one axis and measuring the response along the second axis and (ii) exchanging which axis is compressed and which is measured.

**Fig. 2:**
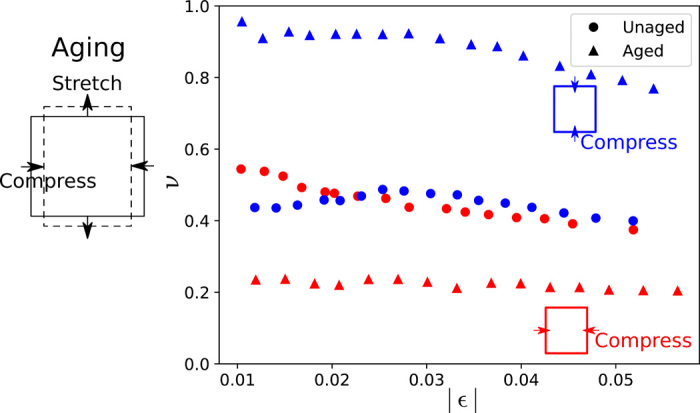
The Poisson’s ratio of experimental foam networks aged under pure shear strain. The abscissa is the strain at which Poisson’s ratio is measured when the network is compressed along a given direction. The unaged (circles) networks do not show a substantial dependence on measurement strain along either axis. The aged networks (triangles) show a marked change in behavior when ν is measured by compressing along the pulling axis (blue) or along the axis that was compressed (red) during aging.

Initially, the Poisson’s ratio of the network measured by compressing the network along either direction gives ν ≈ 0.4. However, once it has been aged under shear, the same measurements give very different results. The Poisson’s ratio drops to ν ≈ 0.2 when compressed in the same direction as the one along which it was aged. However, when compressed along the perpendicular direction, ν increases from ν ≈ 0.4 to ν ≈ 0.8 to 0.9. Figure 2 shows that the material encodes a memory of the direction in which it was aged. Similar anisotropic behavior has been observed in a cross-linked actin network under shear (*24*).

## DISCUSSION

### Generality of directed aging

In all of the examples described so far, aging was used to alter the elastic properties of a network. As a consequence, rearrangements, where a node changes the neighbors with which it is in contact, were not allowed. However, directed aging is not restricted to such situations. It also occurs in contexts in which rearrangements are of crucial importance.

For example, it has been shown that cyclic shearing of a suspension of non-Brownian particles can drive the system toward a state where, under subsequent shearing, each particle simply repeats its former motion (*25*–*29*). This can even be seen in glassy and jammed solids (*30*–*36*). Cyclic driving produces a memory of how the system was prepared. As the cyclic shearing is applied, the training cycles force rearrangements in the particle positions. In another work, lowering of elastic and viscous moduli of a suspension has been observed under shear (*37*).

These systems thus age in a directed fashion as well: They eventually learn how to navigate phase space to produce a periodic response. Each system finds this highly unusual dynamic property by a locally greedy algorithm of nature—the system simply minimizes its energy (or enthalpy) at each time step (in the case of the glassy or jammed solid), or it simply pushes other particles out of the way (in the case of the dilute suspensions). The way in which the material is trained directs the evolution into a nongeneric state.

In another example, it has been shown that an elastic network that restructures in response to periodic forcing can alter its density of states (*38*). In this system, bonds may break or form depending on the interparticle distances and forces. When subjected to periodic forcing, it develops a memory of the frequency, ω, at which it was trained. The periodic forcing thus trains the material to produce an excess density of states at the driving frequency.

**Fig. 3:**
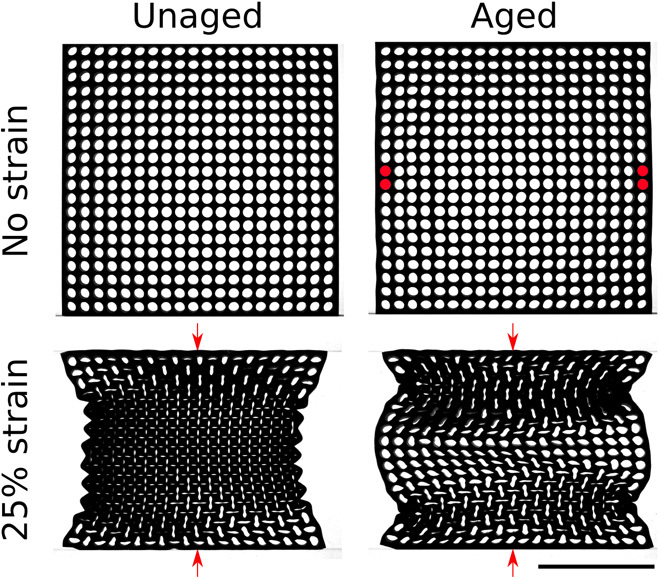
A holey sheet with an array of holes in a square lattice pattern. Initially, the sheet is auxetic under compression along one of the major axes. If the sheet is aged while fixing four holes (shown in red) while the sheet is under compression, the sheet now responds in a scalloped pattern when compressed in the vertical direction. Scale bar, 100 mm.

The idea of directed aging can also be extended to create a localized response to a global distortion. We give one example here. Sheets with a square lattice of circular holes (*39*–*41*) have ν < 0 when compressed along the axis directions (*39*). When this “holey sheet” is compressed, alternating holes distort in perpendicular directions. This ordered collapse results in the transverse edges of the sheet moving closer together, making the sheet auxetic, as shown in Fig. 3.

To obtain a more varied response, we age the sheet in the following way. We plug the holes at the end of the middle two rows so that they retain their circular shape and stay in place as we compress the system along the direction perpendicular to these rows. These rows separate the sheet into two halves that do not communicate with each other. Approximately half of the time that these sheets are compressed, the pattern of holes in one-half is out of phase with those in the other so that the central region does not buckle inward. In these cases, compression along the perpendicular direction creates an overall scallop pattern along the side edges where the sheet juts out in the middle, as shown in Fig. 3.

## CONCLUSIONS

We have demonstrated that aging, which is typically considered to be a detrimental process, can be harnessed to encode a desired elastic response in a material. This directed-aging process relies on incremental changes in stiffness and distortion of the microscopic structure brought about by plastic deformation. We have provided several examples in which directed aging achieves a broad range of responses. We have demonstrated that we can tune the nonlinear as well as the linear response simply by controlling the boundary conditions during the aging process.

The different elastic responses we have discussed have been trained with remarkable ease. Designing elastic properties numerically typically requires a detailed knowledge of the precise interactions, as well as intensive optimization of the parameters. Here, we showed that despite the complex structure of the networks and the nonlinear strains at which they were aged, all that is required is patience while the system memorizes the training conditions and ages toward the target state. This makes our approach well suited to designing materials with unconventional functionality and elastic properties (*11*–*13*, *42*).

**Fig. 4:**
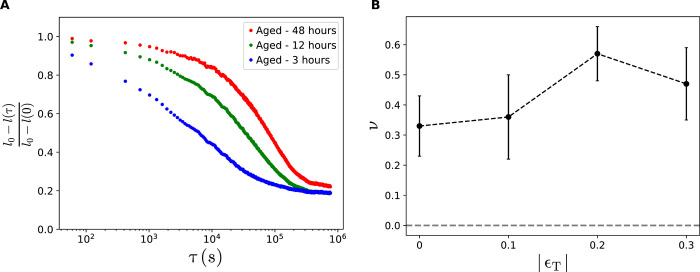
Characterization of EVA foam used. (**A**) Stripes of foam aged under uniaxial compression relax back over time. Relaxation rate depends on how long the sample was aged and the strips do not go back to their original lengths. (**B**) The Poisson’s ratio of solid sheets of foam aged under compression. These sheets do not age the same way as other networks. Instead of a decrease, we see a slight increase in the Poisson’s ratio as a function of aging strain.

Our emphasis in this paper has been to highlight the role of aging alone in creating flexible and unusual functions in materials. However, to realize a design strategy that implements directed aging, we could exploit the strong temperature dependence of the aging process. For example, we could substantially enhance the rate at which desired functionality is acquired by raising the temperature toward the glass transition temperature; lowering the temperature could then freeze in this functionality (*21*, *43*). In addition, we have shown that aging at a higher temperature can also slow, or eliminate, the return to the initial pre-aged state by increasing the irreversible plastic contribution to aging as shown in Fig. 4A for increased training times.

This approach provides an avenue to reach the holy grail of metamaterials—producing them on macroscopic scales. Scaling up metamaterials is usually difficult; if they are designed on a computer, increasing their size increases the required computer resources. Moreover, scaling down the bond size is perhaps even more difficult since it requires a precise control of the detailed structure on small length scales. Directing aging provides a way to alter properties of specific individual bonds by applying only macroscopic strains.

In conclusion, we emphasize that material properties, which are normally considered to be a function of the material composition and structure, can also depend strongly on the history of the imposed deformations. This raises the intriguing possibility that one could use this dependence on aging protocol to read out the history of a material. Could we learn from the elasticity of a rock about geological flows that occurred over millions of years?

## MATERIALS AND METHODS

### Designing networks

All the experimental systems were laser cut from a 0.5″-thick sheet of EVA foam. We used this foam to create a variety of networks for our experiments. To create the jammed packing, we started with a foam sheet and cut out a jammed configuration of discs obtained from a 2D computer simulation. The parts of discs that were overlapping with each other were left undisturbed, and this ensures that we have a fully connected sample. The network designs were derived from a jammed packing. As explained in a previous section, each particle of the jammed packing represents a node of the network, and overlapping pairs of particles are represented by a strut connecting the nodes. For the holey sheets, we again started with a jammed configuration and shrank all the particles uniformly so they do not overlap. We cut holes corresponding to these smaller particles out of a sheet of EVA foam, leaving a sheet with a disordered pattern of holes. The jammed discs and holey sheets explicitly retained the circular nature of a 2D granular packing and were thus closer to the gedanken sandpile experiment discussed in Results. To show that an underlying jammed configuration is not necessary to see this effect, we introduced a fourth system. Starting with a triangular lattice, we allowed the nodes to move by a small amount randomly and then removed bonds at random until the average coordination number of the network was 5.

### Measurement procedure

To measure the Poisson’s ratio of all these systems, we took pictures of the system as we compressed it uniaxially. In these images, we tracked the boundaries of the system. A straight line was fitted to each of the four boundaries, and these lines were used to measure strains in two transverse directions. These measured strains were used to calculate the Poisson’s ratio. All Poisson’s ratios were calculated at an input strain between 2 and 4%.

### Characterization of foam

In this section, we characterized the effect of aging on the EVA foam we used. We determined whether the changes to the foam are permanent or whether the system relaxes to its initial, unaged, state. We measured the time scales on which any relaxation occurs.

To address this, we started by taking strips of foam and compressed them uniaxially by 33%. After letting them age under compression for a fixed amount of time, we removed any external stresses from the strips and measured the change in their length over time. As shown in Fig. 4A, these compressed strips slowly expand, and the rate of expansion depends strongly on the length of time for which they were aged. The strips that were aged the longest are the slowest to expand back. However, we found that even over longer times, the strips do not seem to return to their original length but plateau to a slightly shorter length. This indicates that the changes due to aging have both an irreversible plastic contribution and a viscoelastic relaxation component. In all our experiments, we measured our systems immediately after they have been aged. Measuring the Poisson’s ratio of a single system takes a few minutes, which is much smaller than the relaxation time scales we have measured.

We also characterized the elastic properties of a bulk sheet of foam that has been aged under compression. Figure 4B shows the Poisson’s ratio of a square sheet of foam as a function of aging strain. A solid sheet of foam behaves very differently from all the other systems we have looked at. This shows that the auxetic behavior arising due to directed aging is not a property of the foam but rather is due to the macroscopic structure that is cut out of the foam sheet.

## Supplementary Material

http://advances.sciencemag.org/cgi/content/full/5/12/eaax4215/DC1

Download PDF

Directed aging, memory, and nature’s greed

## SUPPLEMENTARY MATERIALS

Supplementary material for this article is available at http://advances.sciencemag.org/cgi/content/full/5/12/eaax4215/DC1 Evolution of the bulk and shear modulus as a function of time Fig. S1. The evolution of the bulk and shear modulus as a function of time in simulations.
