# Supplementary material for: Directed aging, memory, and nature’s greed
Source: Sci Adv. 2019 Dec 20;5(12):eaax4215. doi: 10.1126/sciadv.aax4215 (PMC6989340; doi:10.1126/sciadv.aax4215)
Supplement: http://advances.sciencemag.org/cgi/content/full/5/12/eaax4215/DC1 [file supp_5_12_eaax4215__index.html]

Science Advances | Science AdvancesAAASSearchScience AdvancesMenu

## Supplementary Materials

**This PDF file includes:**

- Evolution of the bulk and shear modulus as a function of time
- Fig. S1. The evolution of the bulk and shear modulus as a function of time in simulations.

Download PDF

**Files in this Data Supplement:**

- Adobe PDF - aax4215\_SM.pdf
