# Supplementary material for: Directed aging, memory, and nature’s greed
Source: Sci Adv. 2019 Dec 20;5(12):eaax4215. doi: 10.1126/sciadv.aax4215 (PMC6989340; doi:10.1126/sciadv.aax4215)
Supplement: Download PDF [file aax4215_SM.pdf]

Science Advances
AAAS

advances.sciencemag.org/cgi/content/full/5/12/eaax4215/DC1

Supplementary Materials for

Directed aging, memory, and nature's greed

Nidhi Pashine*, Daniel Hexner, Andrea J. Liu, Sidney R. Nagel

*Corresponding author. Email: npashine@uchicago.edu

Published 20 December 2019, *Sci. Adv.* **5**, eaax4215 (2019)
DOI: 10.1126/sciadv.aax4215

This PDF file includes:

Evolution of the bulk and shear modulus as a function of time
Fig. S1. The evolution of the bulk and shear modulus as a function of time in simulations.

Supplementary Materials

Evolution of the bulk and shear modulus as a function of time

We consider how aging affects the elastic moduli in numerical simulations of our model. As the system ages the spring constants become weaker, reducing both the bulk modulus, B, and the shear modulus, G. Since the system is aged under compression, the bulk modulus becomes smaller than the shear modulus (see fig. S1). This results in a negative Poisson's ratio.

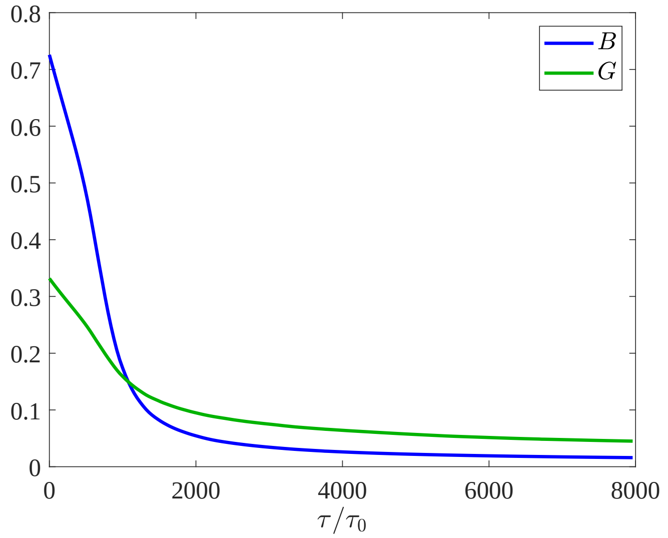

Fig. S1. The evolution of the bulk and shear modulus as a function of time in simulations. Here, the aging strain is the same as in Fig. 1E of the main text, $|\epsilon_T| = 0.025$.
